# Realising sexual and reproductive health and rights of adolescent girls and young women living in slums in Uganda: a qualitative study

**DOI:** 10.1186/s12978-021-01174-z

**Published:** 2021-06-12

**Authors:** Majel McGranahan, Elizabeth Bruno-McClung, Joselyn Nakyeyune, Derrick Aaron Nsibirwa, Christopher Baguma, Christopher Ogwang, Francis Serunjogi, Judith Nakalembe, Marianna Kayaga, Sharifah Sekalala, Oyinlola Oyebode

**Affiliations:** 1 University of Warwick Medical School, Medical School Building, Coventry, UK; 2 Center for Health, Human Rights and Development (CEHURD), Kampala, Uganda; 3 University of Warwick, Law School, Coventry, UK

**Keywords:** Sexual health, Reproductive rights, HIV, Jurisprudence, Patient advocacy, Reproductive Health

## Abstract

**Background:**

Sexual and reproductive health and rights are critical entitlements best supported through human rights-based approaches empowering rights-holders to claim their rights and duty bearers to fulfil their obligations. Implementing these requires information on the current needs and challenges faced by those seeking to claim their sexual and reproductive health and rights. We aimed to identify the underlying factors influencing the realisation of sexual and reproductive health and rights for adolescent girls and young women living Ugandan slums by: (1) exploring the role of relevant service providers and stakeholders; and (2) uncovering knowledge and gaps in protecting adolescent girls’ and young women’s sexual and reproductive health and rights.

**Methods:**

Qualitative data were collected through focus groups and interviews focused on current knowledge, behaviours and attitudes towards sexual and reproductive health and rights among adolescent girls and young women, service providers and stakeholders. Data were analysed thematically using NVivo software. Ten in-depth interviews were conducted with key informants; two focus groups were held with adolescent girls and young women living in two slums in Uganda (21 participants in total); and three focus groups were held with community leaders, service providers, teachers and parents (30 participants in total).

**Results:**

Adolescent girls and young women lacked information regarding their sexual health, services available, and redress mechanisms for rights violations. Formal sources of information were frequently inaccessible. Family members were sometimes the source of rights violations, and informal methods of redressing rights were often sought. Stigma and fear were common features both in healthcare and in the pursuit of formal justice, with duty-bearers habitually breaking confidentiality. Education and training were the predominant suggestions offered for change.

**Conclusions:**

Adolescent girls and young women continue to face obstacles in achieving their full sexual and reproductive health and rights. Targeted interventions for the realisation of adolescent girls’ and young women’s sexual and reproductive health and rights can address underlying causes and positively shift attitudes to promote health.

**Supplementary Information:**

The online version contains supplementary material available at 10.1186/s12978-021-01174-z.

## Plain Language Summary

Every woman is entitled to good sexual and reproductive health. This involves being free from sexually transmitted infections, gender-based violence and maternal mortality, and able to access essential health services. In low and middle-income countries, adolescents comprise a fifth of the population, yet their sexual and reproductive health needs are often overlooked. Adolescent girls and young women are more likely to experience gender-based violence, sexually transmitted infections and poor access to sexual and reproductive health services. Those living in slums have even worse outcomes, but little evidence exists regarding the opportunities and barriers to improving sexual and reproductive health among adolescent girls and young women in a slum setting.

In this study, we conducted focus groups and interviews with 21 adolescent girls and young women living in two Ugandan slums, 10 individuals with an essential role, for example, at state level, as well as 30 other stakeholders including healthcare workers, teachers, parents, district leaders and community support officers working with adolescent girls and young women.

Results indicate that adolescent girls and young women lack information regarding their sexual health, what services are available and who and where to go if they experience violations such as sexual assault. In instances of sexual assault, fear and stigma prevented adolescent girls and young women from going to the police, and instead disputes were handled informally between families.

Participants in our focus groups and interviews felt that education and training were needed, and that health services need to improve. Interventions are necessary to improve adolescent girls’ and young women’s sexual and reproductive health in this setting.

**Supplementary Information:**

The online version contains supplementary material available at 10.1186/s12978-021-01174-z.

## Introduction

Sexual and reproductive health and rights (SRHR) are crucial entitlements relating to women and girls’ sexual and reproductive health [1]. These rights address the prevention of sexually transmitted diseases, including HIV, gender-based violence, maternal mortality and provision of essential health services [2–4]. Since the International Conference on Population and Development (1994) and the Beijing Platform for Action (1995), national SRHR policies have been demonstrated to support societies and contribute to a country’s wealth [2, 3, 5, 6]. The importance of SRHR is further underscored by section 5.6 of Sustainable Development Goal 5, dedicated to achieving SRHR for all [7].

Human rights-based approaches (HRBA), by emphasizing rights over needs, have become the focus of sustainable strategies for change [5, 8, 9]. The basic principles of HRBA include empowerment of rights-holders and duty-bearers (those responsible for protecting and enacting human rights), non-discrimination, open participation, accountability, and defined and established linkages between rights-holders and duty-bearers [5, 8]. For sexual and reproductive health, HRBA empower women to claim their rights and duty-bearers to fulfil their obligations [8, 10]. By focusing on non-discrimination, HRBA also specifically consider those who are vulnerable, marginalized and discriminated against [8, 10], giving agency to the less powerful in society. Achieving these principles requires coordinated, multi-sectorial approaches, based on an analytical understanding of the needs of groups, available resources and challenges [11, 12].

Adolescents and emerging adults aged 15–24 comprise around a fifth of the population in low and middle-income countries (LMICs) [13], yet their needs are often overlooked and, particularly regarding sexual and reproductive health, underfunded [2, 6, 14]. In Uganda, teenage pregnancy rates are high, with one in four women aged 15–19 giving birth [15]. Adolescent girls and young women (AGYW) experience higher risks of gender-based violence, a disproportionate likelihood of sexually transmitted diseases, and frequently lack access to sexual and reproductive health services [2, 6, 16]. For AGYW living in urban slums, their visibility is reduced further by the double vulnerability of age and poverty [17].

Previous studies considering SRHR of women in LMICs have focused predominantly on sex workers, or maternity settings [18] A recent scoping review identified no qualitative evidence regarding sexual and reproductive health challenges among young people living in slums in Uganda, and limited qualitative evidence in the rest of Sub-Saharan Africa [17]. A cross-sectional study undertaken among 13–24 year olds in Makindye and Nakawa Divisions of Kampala, Uganda, identified sexual abuse a significant issue among participants, but the underlying facilitators and barriers were not explored [19].

There remains a need for studies focusing on AGYW, regardless of parity or engagement in sex work. This study aimed to explore the underlying factors impacting on the realisation of SRHR of AGYW living in slums in Wakiso District, Uganda.

## Methods

In-depth interviews and focus groups were conducted in Wakiso District, Uganda, between February-August 2019. Qualitative data were collected regarding knowledge, attitudes and experiences of SRHR of AGYW living within slums. Kibwa and Kileku slums were selected as both are unplanned settlements representing established (Kibwa) and emerging (Kileku) slums whose semi-permanent population is known locally to face overcrowding, unhygienic conditions and high levels of violence and sexual crimes.

Two focus groups (one each in Kileku and Kibwa) were held with AGYW aged 14–23 years resident in either slum, recruited through established community connections. There were no exclusion criteria as a wide range of life-experiences were sought. Invitations to participate were by phone or verbal invitation. Focus groups were limited to an hour, during daytime, in private and accessible locations.

Ten 1:1 in-depth interviews were held with key informants including state and non-state actors (Table 1). Purposive sampling, with assistance from the Wakiso District community development officer, was used to select participants based on their essential role in realising AGYW’s SRHR. Participants were invited to participate by telephone.

**Table 1:** Study participants

	Description	Kibwa or Kileku	Description of facilitator
Focus Group 1	10 participants: Female Aged 14–23 years	Living in Kibwa slum	2 × facilitators 2 × note takers
Focus Group 2	11 participants: Female Aged 16–20 years	Living in Kileku slum	2 × facilitators 2 × note takers
Focus Group 3	10 participants: Male and female Local leaders Local chairperson Youth council representatives	Working in Kileku slum	1 × facilitator 2 × note takers
Focus Group 4	10 participants: Male and female Local leaders Local chairperson Youth council representatives	Working in Kibwa slum	1 × facilitator 2 × note takers
Focus Group 5	10 participants: Male and female Health workers (Public hospital) Pharmacists (Private) School teachers Parents	Working in Kibwa slum	1 × facilitator 2 × note takers
One to one interviews (× 10)	10 participants: Male and female 3 district leaders 7 staff from community-based organisations (3 community outreach personnel and 4 team leaders)	Working in Kibwa and/or Kileku slums	1 interviewer per participant

Three focus groups were held with stakeholders, selected for their obligations to SRHR for AGYW (Table 1). Purposive sampling, with assistance from the district officer for health, and snowball sampling were used. Invitations were by telephone, email and letters. Groups were held in private spaces separate to working environments.

Participants gave written informed consent. Participants under 18 years who were unmarried required a caregiver’s consent, unless pregnant.

Individuals who participated in focus groups were each given a number to identify themselves with (between 1 and 11), so individual’s names were not used in recordings. No prior relationship existed between research team members and participants. Ethics committees in the UK and Uganda approved the study design.

### Data collection

Interview and focus group topic guides were developed by members of Center for Health, Human Rights and Development (CEHURD), Uganda and University of Warwick, UK (see Additional files 1, 2 and 3).

Trained researchers from CEHURD with qualitative research experience facilitated each focus group. Two additional CEHURD members wrote reflective notes. Focus groups were held in Luganda, with English as required.

Data were recorded, transcribed, translated and checked for accuracy by members of CEHURD. Triangulation with reflective notes was undertaken to reduce researcher bias.

### Data analysis

Data were coded by members of CEHURD (DAN, JN, FA) and University of Warwick (MM, EB-M) using NVIVO with coding decisions discussed with a third University of Warwick team member (OO). Transcripts were thematically analysed using simultaneous inductive and deductive approaches with emerging themes compared within and across transcripts. Disputes were handled through team discussions and consensus.

Findings were reported following the Standards for Reporting Qualitative Research [20].

### Patient and public involvement

Patients and/or public were not involved in the design, recruitment, conduct, or reporting of this study.

## Results

Of 61 people invited to take part in the study, none declined. Table 1 describes the participant characteristics. AGYW included in the study were aged 14–23 years; six participants were aged 14–17 years, eleven were aged 18–20 years and four were aged 21–23 years. Table 2 outlines the identified themes and sub-themes. Additional file 4 includes examples of illustrative quotes for each sub-theme.

**Table 2:** Themes and sub-themes identified

Theme	Sub-theme
Understanding of Sexual Health including sexually transmitted infections and HIV	Myths and misconceptions Knowledge of HIV among adolescent girls and young women (AGWY) Barriers to education provision
Understanding of Sexual and Reproductive Health and Rights (SRHR)	Understanding of consent Understanding of health and rights Lack of knowledge about SRHR Lack of knowledge preventing redress of rights Lack of knowledge as a barrier to healthcare Understanding of SRHR by stakeholders Sources of knowledge for AGWY Sources of knowledge for stakeholders
Sources of support	Support whilst menstruating or sick Disintegration of family system of support Role of peers Source of support in case of harassment Where to go for justice
Experience of healthcare	Respect needed to improve healthcare Positive experience of healthcare Needs unrecognised among AGWY Different treatment if poor Use of non-traditional medicine due to fear or cost Lack of resources or medications Lack of psychological support Poor service at hospital
Age, maturity and legal age	Age at maturity Being treated differently due to age Stigma due to age Education needed to support young people
Violations of rights and context	People with money are treated differently Attempted corruption Violations within healthcare Breach of confidentiality Stigma associated with violation Normalisation of sexual assault Taking advantage of AGYW Poverty as a driver for rights violations Power imbalance as a driver for rights violations
Barriers to justice	Barrier to justice Prioritising reputation Barrier to justice: Cost/corruption Experiences of corruption Barrier to justice: Stigma Health professionals not testifying following rape Threats from violators Concern from victim they will be left without support Police not doing their job properly Only school attendees taken seriously
Role of parents	Parents forcing child marriage Parents do not believe in family planning Parents not open with children Belief that children will copy parents’ behaviours Home environment as a driver for violation Money as a driver for child marriage
Services available	Services available for HIV Services available from interviewees Traditional healers
Barriers to healthcare access	Lack of husband’s presence as a barrier to access Fear as a barrier to healthcare Distance as a barrier to healthcare Stigma as a barrier to access Husband as barrier to access Parents as a barrier to access Cost as a barrier to access Language as a barrier Rumours as a barrier to care
Consequences of pregnancy	Thrown out due to pregnancy School dropout due to pregnancy
Drivers for violations	Alcohol and drugs as driver for violations Belief that AGWY dressed inappropriately Money as a driver for exploitation
Redress of rights and challenges	Informal redress of rights Delay in redress Length of sentence felt to be too short
Sources of information regarding SRHR and services and areas for improvement / challenge	How AGWY know about service availability Need to educate boys as well as girls Misconceptions among stakeholders Resistance to learning among AGYW Slum context for learning Feelings that AGYW will not do as told Need for education Need to advertise services more
Changes needed to allow redress of rights	Need for empowerment Government needs to change policy/law
Suggestions for services	Training for stakeholders More funding Stop corruption Increase service availability Empower women

### Understanding of sexual health and rights

AGYW had a reasonable understanding of HIV: identifying how it is spread, life-long implications of diagnosis and the importance of medication adherence. Several key informants identified misconceptions about sexual health held by AGYW.

“But they are fed the information like if you use a condom, it will get stuck inside you, you understand.” Community Support Officer 1.

Key informants stated that lack of education on SRHR was a reason for misconceptions. Many key informants believed that AGYW underutilized healthcare services because they were unaware of them or what they offered.

“…they don’t keep informing the community of the availability of these services and the fact that Wakiso is a peri-urban district, people settle and people go away, people settle and people go away. So the people who come in tend to miss the services thinking they are not available.” District Leader 1.

Key informants were able to name some SRHR. AGYW articulated their right to consent to sexual intimacy, access health services and accept healthcare. Key informants and stakeholders felt AGYW lacked information on how to redress their rights if violated, which was borne out by testimony from AGYW suggesting they were unsure where to go if their rights were violated.

“they can get justice, but they haven’t got guidance. They even say, am pregnant and am 13 years but if they are going to imprison [my partner], I should also be imprisoned. They don’t understand that we are helping them in their lives to save them and also from diseases.” Number 2, Focus Group 4.

### Sources of information

A wide range of sources provided information about SRHR for stakeholders and key informants, for example training workshops and visiting lawyers. However, government restrictions on reproductive health education were seen as a significant barrier for AGYW.

“They have the right but our government and leaders like the religious leaders currently don’t accept or allow these young girls to know about some health issues for example: reproductive health.” Community Support Officer 2.

Official means of disseminating information were through radio programs and fliers/posters in English. Some stakeholders noted that printing information in English was not helpful to those who could not read or speak English.

AGYW identified their peers and community-based organisations as predominant informal information sources. Parents were often seen as barriers to understanding: withholding reproductive health information. Stakeholders described parents as unsupportive of family planning and lacking openness with their children about sexual health.

“There are parents that don’t believe in family planning. They come and say, my child has started taking medication. It’s also a problem especially because the parents also don’t know about the importance of family planning” Number 4, Focus Group 4.

“But most parents keep things secret from their children and we don’t tell them what is going on in the world.” Number 5, Focus Group 3.

### Experience of and barriers to healthcare

Nearly all participants stated that local healthcare services lacked resources and medications. AGYW were therefore asked to pay for healthcare: from fee-for-service clinics to essential medications. For many, the financial burden was too high. Combined with fear and stigma, some participants stated that AGYW often resort to using unregulated and traditional medicine.

“…when I reach the pharmacy where am to buy the drugs from, the drugs are 30,000ugx and I only have 10,000ugx in my bag, so I find that medication too expensive. In that case, I will just go and pluck some “omululuza” and “kamunye” and I drink because it is the cheaper option that will help me.” Number 10, Focus Group 5.

“They fear if they get a problem may be they want to abort, they can use these herbs and someone calls you and says this one is here dying, they say she has taken herbs.” Community Support Officer 3.

Stories of poor healthcare and stigma associated with sexual health issues were common reasons for AGYW not to utilize services. Many AGYW felt they were treated differently because they were young and poor, and those in duty-bearer positions did not listen to their needs. For AGYW accessing healthcare, perceived lack of respect caused them to shy away from further healthcare. Some AGYW recalled positive healthcare experiences.

“I was given a lot of care where I gave birth from even when I hadn’t paid money but that was in the [name omitted] government hospital.” Number 10, Focus Group 2.

Some AGYW were denied healthcare access when visiting without a husband present. A key informant stated husbands often prevented their wives from accessing family planning services.

AGYW and key informants stated that despite the peri-urban environment, travel distance and costs were significant barriers to healthcare.

““…the distance is long and it is not like everyone can walk there or has the money…” Number 10, Focus Group 1.

### Violations of rights

Most participants specified that money and poverty played a significant role in the violation of AGYW’s rights. Participants recalled stories of desperate family members for whom money was a driver for them facilitating their daughters to engage in underage sex and child marriage. However, parents’ actions were not always described as seeking monetary gain. A local leader noted that parents forced their children to marry because it mirrored their own experience.

“…our problem is with the parents who force their children to get married fast mainly because the parents too got married early.” Number 5, Focus Group 4.

AGYW also described experiences where perpetrators with money were treated differently, and a perpetrator’s word was considered more credible than the word of assaulted AGYW. Other AGWY described experiences of attempted corruption of their rights for money.

“My uncle came home and he was kind of forcing me to accept money from him which was 500,000shs and placed it on the table asking me to get the money and testify in court saying I wanted what happened to me.” Number 4, Focus Group 2.

Many AGYW described guardians and duty-bearers violating their rights. This was echoed by stakeholders and included confidentiality breaches: from teachers to health workers.

“What has brought the biggest issue is health worker, they don’t keep confidentiality. So people will not go to see them if they don’t keep confidentiality.” Number 7, Focus Group 4.

Key informants noted that sexual assault violations were normalized over a certain age. Some participants stated that AGYW are taken advantage of when trying to better their education or obtain jobs. High alcohol and drug use amongst men were named as underlying causes of domestic violence towards women.

“Sexual domestic violence is high in areas like this. For us men, the rate of drinking alcohol and drug abuse—you know what these result into. When they go back home, they harass women, because of poverty and other things. So our sisters have got problems because domestic violence is high and rape.” Unknown Number, Focus Group 3.

### Barriers to justice and redress of rights violations

Many stakeholders noted that while official channels existed for women to redress their rights, cases were often informally handled between families. These ‘community courts’ left little support for women involved.

“The other challenge that we have is that parents also always negotiate with the people that rape their children and we find ourselves in situations where cases are settled between families without consideration of the challenges that the survivor is going through or even worse still the diseases she may contract from such experiences.” Number 5, Focus Group 4.

Even when attempting to use formal channels of justice, several AGYW experienced the prioritizing of other’s reputation over their right to justice. Guardians in the home, education, and healthcare environment suppressed their rights due to appearances and to save perpetrators from jail.

“But when I tried to tell the owner of the school, he told me to leave that alone and kept silent. [He said] “The good thing you are soon finishing senior 4 and you leave the school, leave that alone, because if you report, you are going to tarnish the school’s reputation.”” Number 4, Focus Group 1.

“But when she told the person she stays with, that is her grandmother, the mother of her uncle, she asked one thing, “Do you want my son to be arrested?” so after they had raped her, she was in pain, but because her grandmother doesn’t want her son to be arrested she didn’t get her.” Number 10, Focus Group 1.

AGYW sometimes choose to live without justice because they fear they will be left without support.

“So someone may not get justice because the man used her and that if the man is arrested, she may not get supports and there will be no one to support her. So it ends that it is like they have not got justice because she wants to find a way of ensuring the man looks after her.” Number 9, Focus Group 3.

Key informants identified stigma towards AGYW who experienced violations, and AGYW felt stigmatised when pregnant.

“Men stigmatizing us throwing comments such as look at her she gave birth at a young age look at her legs those are the comments they throw at us.” Number 4, Focus Group 2.

Stigma was related to experiencing violations and the act of redressing it. Some participants stated that where violations were reported, the victim endured further discrimination and shame. The potential for additional stigma associated with reporting violations presented a significant barrier.

“Then the shame that is attached to the person who had been violated, oh dear, society will be like “So she thought that reporting will help?” Everywhere she will pass, she will be despised the more.” Community Support Officer 4.

An individual’s status in terms of money, age, and education greatly influenced whether a reported violation was believed or taken seriously. Cost and corruption were a common theme in preventing justice, particularly regarding police involvement. Many participants stated they felt the police were not effective and were open to corruption.

“Our side at the police, our mother had 100,000 ugx but she told them she had 50,000ugx, then they agrees to come and arrest the person. But we waited for them and they didn’t show. When we went back, they told us we must add more money because what we had given them was less/little, so we abandoned the case.” Number 6, Focus Group 1.

Health professionals were seen as reluctant to testify or support victims coming forward.

### Suggestions for change

Many participants suggested that further education would empower AGYW to speak up against violations. Also mentioned was the need to educate other stakeholders, members of the community and government officials, as misconceptions were evident at all levels. One participant suggested that SRHR education should extend to boys and men given the importance of their role in ensuring women achieve their SRHR.

“So I think the other challenge is sexual and reproductive health rights being looked at as exclusively for girls, so the boys will always do whatever they want thinking that this is only for girls, which to me is something the society needs to come out strongly to educate the male youths.” Community Support Officer 4.

One service provider, however, felt that education may be wasted on AGYW. They felt a divide between adults and the younger generation and suggested that the younger generation resisted advice from elders.

“Most don’t want to be sensitized. In that even though you organize a training, they do not attend and yet they would have been of use to them in learning and understanding if there is any chance of an issue like this happening in the future and it is in these trainings where they would get knowledge on what to do in case the need arises.” Number 6, Focus Group 5.

According to key informants and stakeholders, there may be little difference until the laws are changed to align with AGYW’s needs even with increased education.

“They [young women] may want to acquire post abortion or abortion services but it’s against the law so they don’t, maybe some of these cases end up into death.” Community Support Officer 5.

“For example in schools the information we give them according to the Ministry of Education and Sports, it is less to what they need They tell us not to talk about abortion but this is what the girls are facing and they need this information and service provision somehow but they can’t access. They need the knowledge but you still can’t give because it’s against the law.” Community Support Officer 5.

Another key informant suggested that more resources and funding specifically for adolescent health would improve service delivery.

“More funding should be directed towards Adolescent health because we really don’t have a vote for adolescent health as a standalone. We are running… integrating it with other services and we run on other service now like HIV and ride on them.” District Leader 2.

Many stakeholders suggested that fighting corruption would make a difference to overall SRHR service delivery. One participant felt sentences for perpetrators were too light, and did not act as significant disincentives to repeat offence.

“Can I also add that the police is not doing us enough justice because ideally whoever violates should be handled by a strong law, people don’t understand that once you violate a girl’s or boy’s sexual rights you are ruining this person for good. Someone found guilty takes a very light sentence and goes and violates another one, they bring them back and it’s the same light sentence.” Community Support Officer 4.

## Discussion

Themes arising in the data describe SRHR gaps and violations as they relate to AGYW living in urban slums in Uganda, and suggest areas amenable to intervention based on HRBA principles.

Analysis revealed that poverty remains a common driver of SRHR violations: reducing healthcare access, rendering treatments unaffordable, exposing vulnerabilities in the lives of AGYW and their families, and preventing justice by tolerating environments where corruption, particularly by police, is pervasive. Despite these challenges, there appeared ample opportunity for promoting empowerment through education, as recommended by the World Health Organisation [21]. With knowledge gaps identified, raising awareness amongst AGYW, stakeholders and the wider community could address misconceptions, encourage participation and set the stage for stronger accountability mechanisms.

Our study is the first to outline the challenges faced by AGYW in achieving SRHR in a slum setting in Uganda. Indeed, a recent scoping review identified no qualitative evidence regarding sexual and reproductive health challenges among young people living in slums in Uganda, and limited qualitative evidence in the rest of Sub-Saharan Africa [17]. A cross-sectional study undertaken among 13–24 year olds in Makindye and Nakawa Divisions of Kampala identified sexual assault as a common issue, in keeping with our findings [19].

Initiatives like specifically criminalizing gender-based violence, as enacted in Uganda in 2010 [22], can be important in the journey towards supporting AGYW to achieve SRHR. However, as data from this study show, legislation alone cannot solve the problem. Other qualitative and implementation studies have utilized bottom-up HRBA to affect change. Methods focusing on legal empowerment for health promotion have been applied to HIV [23], while community-based awareness campaigns have demonstrated attitude shifts towards young mothers through empowerment and income generation [24]. These interventions highlight the strength in strategically incorporating the wider community when addressing SRHR issues.

This study encouraged open discussions of experiences and opinions amongst peers. Critical to the process of developing HRBAs [11], this information provides a current assessment and analysis of rights, upon which interventions can be tailored to address the specific causes of non-realisation of rights for these AGYW, thus avoiding carbon copying of methods elsewhere and strengthening the likelihood of sustainability [25].

The UNFPA suggests that empowerment comes from not only knowing your rights but also knowing that those in power are aware, duty-bound, and actively supporting these rights [8]. An additional strength of this study is in establishing partnerships and networking stakeholders and duty-bearers in their awareness and commitment to protection of AGYW’s SRHR. The interviews with key informants brought additional depth, painting a wider picture of the structural causes effecting the realisation of AGYW’s SRHR.

This study was conducted in just two slums in a single country and not all findings will be generalisable to other contexts. Other limitations include the self-selecting nature of participants, particularly AGYW who were under 18. Requiring parents’ consent may have limited participation to those whose parents were open and might not represent experiences of AGYW whose parents held different attitudes. However, this was likely overcome with the inclusion of AGYW over 18; in fact, parental beliefs and behaviour as rights violations and barriers to justice were identified. The participant threshold was predetermined based on available time and resources, and on participants’ roles and affiliations within the slums. Further studies in urban areas allowing for more participants would help achieve data saturation.

## Conclusions

This study has identified important barriers and facilitators for AGYW to achieve their SRHR in an urban slum context. Barriers identified included stigma towards pregnant teenagers, cost preventing healthcare access, and family, police and school sometimes acting as barriers to the redress of rights. Facilitators identified included education to address misconceptions and improved accountability mechanisms. The knowledge obtained from this study and the connections established will be used to develop an intervention based on legal empowerment and social accountability approaches. Grounded in human rights norms, the resulting intervention could increase awareness, empower and promote agency in AGYW living in slums [25]. The need for such an intervention was evident in discussions with key informants, many of whom actively sought advice regarding rights-based implementation from the research team. While this study was conducted in Uganda, worldwide reports suggest that many countries are struggling with similar misinformation, resource constraints and community-driven stigmas [2], suggesting that a resulting intervention may be relevant to other contexts too. Through awareness and continued engagement, targeted interventions for the realisation of AGYW’s SRHR can address underlying causes and positively shift attitudes for the promotion of health.

## Supplementary Information

**Additional file 1.** Is the topic guide for interviews.

**Additional file 2.** Is the topic guide for stakeholder focus groups.

**Additional file 3.** Is the topic guide for AGYW focus groups.

**Additional file 4.** Is a Table outlining themes and sub-themes identified with illustrative quotes.

## Data Availability

The anonymised datasets used and/or analysed during the current study are available from the corresponding author on reasonable request.

## Abbreviations

AGYW: Adolescent girls and young women
SRHR: Sexual and reproductive health and rights
HRBA: Human rights-based approach
CEHURD: Center for health, human rights and development, Uganda
LMIC: Low and middle-income countries
HIV: Human immunodeficiency virus
