# Supplementary material for: Realising sexual and reproductive health and rights of adolescent girls and young women living in slums in Uganda: a qualitative study
Source: Reprod Health. 2021 Jun 12;18:125. doi: 10.1186/s12978-021-01174-z (PMC8199558; doi:10.1186/s12978-021-01174-z)
Supplement: Supplementary file 1 — Additional file 1. Is the topic guide for interviews. [file 12978_2021_1174_MOESM1_ESM.doc]

**
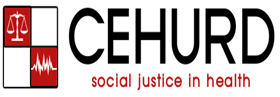
**

**Integrating Legal Empowerment and Social Accountability (LESA) for Sexual Reproductive Health (SRH) and HIV services for young people in selected slums Areas in Uganda**

**TOOL:** Individual interviews with key informant interviews

| Information needs | This tool will help us map the knowledge of district leaders/key informants/AGYW/organizations working with AGYW on rights and practices advancing sexual and reproductive health rights and HIV services for young women. |
| --- | --- |
| Source | - District leaders - Key informants |

| **KII Characteristics** | | | |
| --- | --- | --- | --- |
| Facilitators Name |  | Date of Interview |  |
| Respondents Pseudo Name |  | Position |  |
| Respondents gender |  | Time started the interview |  |
|  |  | Time ended the interview |  |
| Venue of interview |  | | |

| Themes | **Questions** |
| --- | --- |
| Introduction | My name is_____, I will be interviewing you. |
|  | Good day, I am representing Center for Health, Human Rights and Development (CEHURD) to conduct a study to map the knowledge of girls and young women on sexual and reproductive health and HIV services.  This project aims to help girls and young women living in slums in Uganda to achieve their sexual and reproductive rights by exploring their rights under the Domestic Violence Act of 2009. We are reaching out to you because you are a key decision maker in sexual reproductive health service delivery for girls and young women in this community and may be aware of the challenges in achieving their rights. The opinions and experiences you tell us about will be used to study how increasing knowledge and understanding of the law, leads to changes in attitude and behaviour to protect health.  Participation is entirely voluntary, and a decision to withdraw from the study without giving a reason, will not affect you in any way. If you wish to withdraw during the interview, please simply inform the interviewer and you will be free to leave. You are also free to decline to answer any questions posed during the interview, without withdrawing from the discussion completely.  The study findings will be reported in a research report which will be presented at a workshop with district officials and other people who make policy and deliver services for young people. We will also publish our findings in a scientific journal. We will work with local organisations to make people aware of what we find out through this research project.  I will now start the recording if you give me permission to continue with this interview..? (Pause)  (1) Yes; assent obtain (2) No (assent NOT obtained) [DELAY RECORDING, ANSWER ANY QUESTIONS OR ALLOW PARTICIPANT TO WITHDRAW] |
|  | What do you know about SRHR? |
| 1.a | Can you mention some of the SRHR services? |
|  | Are any of the above mentioned services available for AGYW in your community? |
| 2.a | In your opinion, do these services meet the set standards in terms of?  Availability  Accessibility  Affordability  Quality |
| 2.b | Can you justify your answers in 2a above?  Availability  Accessibility  Affordability  Quality |
| 3 | How would you describe the magnitude of the HIV/SRH challenges in this district, and how do the district indicators *generally* compare with the national indicators? |
| 3.a | What population groups in this district are most affected by HIV? |
| 3.b | What special challenges – if any – do AGYW face in this district that might make them more vulnerable to HIV/SRHR challenges? |
| 3.d | Are HIV service centers in this district well equipped to adequately serve AGYW (including survivors of sexual and gender based violence) who need HIV prevention and care services? |
| 3.e | Are there any special provisions that have been made to cater for AGYW? |
| 3.f | Are health facilities, especially HIV service points, adolescent/youth-friendly? |
| 4 | What is the policy and progress on implementing the following services in this district:  Safe male circumcision for HIV prevention?  Post-exposure prophylaxis (PEP); is PEP accessible to GBV survivors? What is the procedure for accessing it? (E.g. does a woman need to have made a police report?)Pre-exposure prophylaxis (PrEP)?  “Test and treat” policy for HIV?  Emergency contraception? |
| 4.a | What challenges does the health sector face in reaching AGYW with HIV prevention and SRH services? |
| 5 | Where do you think improvements need to be made if AGYW are to access quality HIV services at the community level? |
| 5.a | As far as the provision of SRHR/HIV services, how can the following stakeholder & duty bearers do to enhance service access by AGYW;  Health workers  Health Unit Management Committees  Village Health Teams/Community Health Extension Workers  Village HIV committees  Community-based organizations  Schools  Police and other law enforcement organs (e.g. LCs)  District Local Government  Ministry of Health |
| 6 | I request you explain to me what the mandate of your position is. |
| 6.a | What is the role of your office in the response to SRH challenges including HIV amongst AGYW in this district? |
| 7 | Do any bylaws exist to protect adolescent girls and young women from sexual assault? |
| 7.a | If so, what are they? |
| 7.b | If not, do you believe that there should be? |
| 7.c | Have adolescent girls or young women ever come to you to report a case of sexual assault?  If so, how did you deal with it? |
| 7.d | In your opinion, who empowers women to come to stakeholders/duty bearers like you when they wish to report a case of sexual assault?  Examples: Mother? Father? Friends? |

**We have come to end of this discussion; we thank you for taking time to participate in this interview.**
