# Supplementary material for: Realising sexual and reproductive health and rights of adolescent girls and young women living in slums in Uganda: a qualitative study
Source: Reprod Health. 2021 Jun 12;18:125. doi: 10.1186/s12978-021-01174-z (PMC8199558; doi:10.1186/s12978-021-01174-z)
Supplement: Supplementary file 2 — Additional file 2. Is the topic guide for stakeholder focus groups. [file 12978_2021_1174_MOESM2_ESM.doc]

**
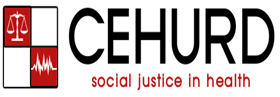
**

**Integrating Legal Empowerment and Social Accountability (LESA) for Sexual Reproductive Health (SRH) and HIV services for young people in selected slums Areas in Uganda**

**TOOL:** Individual interviews with key organizations working with young women

| Information needs | This tool will help us map the knowledge of district leaders/key informants/AGYW/organization working with AGYW on rights and practices advancing sexual and reproductive health rights and HIV services for young women. |
| --- | --- |
| Source | - Organizations working with AGYW |

| **KII Characteristics** | | | |
| --- | --- | --- | --- |
| Facilitators Name |  | Date of FGD |  |
|  |  | Time started the FGD |  |
|  |  | Time ended the FGD |  |
| Venue of FGD |  | | |

| Themes | **Questions** |
| --- | --- |
| Introduction | My name is_____, I will be facilitating the discussion today. |
|  | Good day, I am representing Center for Health, Human Rights and Development (CEHURD) to conduct a study to map the knowledge of girls and young women on sexual and reproductive health and HIV services.  This project aims to help girls and young women living in slums in Uganda to achieve their sexual and reproductive rights by exploring their rights under the Domestic Violence Act of 2009. We are reaching out to you as you have a role to play in supporting girls and young women and you might have experience that can help us understand the challenges regarding their sexual and reproductive health. The opinions and experiences you tell us about will be used to study how increasing knowledge and understanding of the law, leads to changes in attitude and behaviour to protect health.  The study findings will be reported in a research report which will be presented at a workshop with district officials and other people who make policy and deliver services for young people. We will also publish our findings in a scientific journal. We will work with local organisations to make people aware of what we find out through this research project. Our hope is that we can use this new understanding to improve sexual and reproductive health in young women.  You may choose to participate in this study or not and you may withdraw from this discussion at any time. You have each completed a participant consent form indicating that you consent to participating in this focus group.  Before we begin I would like us to agree that we will keep the information disclosed during this discussion confidential.  I will now start the recording if you give me permission to continue with this interview..? (Pause)  (1) Yes; assent obtain (2) No (assent NOT obtained) [DELAY RECORDING, ANSWER ANY QUESTIONS OR ALLOW PARTICIPANT TO WITHDRAW] |
|  | Do you know about rights to Sexual Reproductive Health? |
| 1.a | Can you mention some of these rights |
| 1.b | How did you get to know about these rights? |
| 2 | In your opinion, what are the challenges faced by AGYW in accessing SRHR services in this community? |
| 3 | In your opinion, what are the health rights violations faced by AGYW in this community? |
| 4a | Are there any existing redress mechanisms for the violations at community/district level? |
| 4.b | If yes, What are some of these mechanisms? |
| 4.c | In your opinion, can AGYW access justice in case of violations? |
| 4.d | If yes, to what extent can they access justice in cases of violations? |
| 5.a | Are there any SRH/HIV service delivery challenges that AGYW face in this community? |
| 5.b | If yes, can you mention some of the SRHR /HIV service delivery challenges faced by AGYW in this community? |
| 6. | In your opinion, what are some of the recommendations that you can suggest to improve the delivery of SRH/HIV services for AGYW? |

**We have come to end of this discussion; we thank you for taking time to participate in this interview.**

**END**
