# Supplementary material for: Realising sexual and reproductive health and rights of adolescent girls and young women living in slums in Uganda: a qualitative study
Source: Reprod Health. 2021 Jun 12;18:125. doi: 10.1186/s12978-021-01174-z (PMC8199558; doi:10.1186/s12978-021-01174-z)
Supplement: Supplementary file 3 — Additional file 3. Is the topic guide for AGYW focus groups. [file 12978_2021_1174_MOESM3_ESM.doc]

**
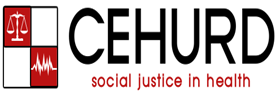
**

**Integrating Legal Empowerment and Social Accountability (LESA) for Sexual Reproductive Health (SRH) and HIV services for young people in selected slums Areas in Uganda**

**TOOL:** Focus Group Discussion for Adolescent Girls and Young Women

| Information needs | This tool will help us map the knowledge of key informants/district leaders/AGYW/organizations working with AGYW on rights and practices advancing sexual and reproductive health rights and HIV services for young women. |
| --- | --- |
| Source | - AGYW |

| **KII Characteristics** | | | |
| --- | --- | --- | --- |
| Facilitators Name |  | Date of FGD |  |
|  |  | Position |  |
|  |  | Time started the FGD |  |
|  |  | Time ended the FGD |  |
| Venue of FGD |  | | |

| Themes | **Questions** |
| --- | --- |
| Introduction | My name is_____, I will be facilitating the discussion today. Would you like to share your names? |
|  | Good day, I am representing Center for Health, Human Rights and Development (CEHURD) to conduct a study to map the knowledge of girls and young women on sexual and reproductive health and HIV services.  I want to know more about the sexual and reproductive health and HIV services for girls and young women in this community as part of the study. This project aims to help girls and young women living in slums in Uganda to achieve their sexual and reproductive rights by exploring their rights under the Domestic Violence Act of 2009. We are reaching out to you as you are girls and young women and you might have experience that can help us understand the challenges and needs that you have regarding your sexual and reproductive health, or you may know of others’ experience. The opinions and experiences you tell us about will be used to study how increasing knowledge and understanding of the law, leads to changes in attitude and behaviour to protect health.  Participation is entirely voluntary, and a decision to withdraw from the study without giving a reason, will not affect you in any way. The information we collect will only be seen by our small research team at CEHURD and your names and identities will be removed from any data shared with University of Warwick and from our research findings when they are presented.  The study findings will be reported in a research report which will be presented at a workshop with district officials and other people who make policy and deliver services for young people. We will also publish our findings in a scientific journal. We will work with local organisations to make people aware of what we find out through this research project. Our hope is that this will help to improve sexual and reproductive health in young women in Uganda.  Before we begin I would like us to agree as a group, that nothing that a participant brings up within these walls will be discussed with someone who is not in this group. We all have to keep the information disclosed during this discussion confidential.  I will now start the recording unless anyone raises their hand..? (Pause)  (1) No hands raised, assent obtain (2) Hands raised (assent NOT obtained) [DELAY RECORDING, ANSWER ANY QUESTIONS OR ALLOW PARTICIPANT TO WITHDRAW] |
|  | What do you know about your Rights to Sexual and Reproductive Health (SRHR)? |
| 1a. | How did you get to know about these rights? |
| 1.b | Can any of you name for me some of the rights? |
|  | Have any of you ever demanded for these services? |
| 2.a | If Yes, Where have you demand for them to be fulfilled? |
|  | In your views, do health centers and health practitioners observe the SRHR of Adolescent Girls and Young Women (AGYW)? |
| 3a. | Can any of you please share your experience in accessing SRH services in your community? |
| 3b. | What challenges have any of you faced in receiving or demanding for these rights? |
| 3.c | What recommendations would any of you give to the people responsible? |
| **HIV Services** | |
| 4. | - What can you tell me about HIV? |
| 4a. | - Do HIV services exist for your community? |
| 4c. | Can any of you tell me how you got to know about these services? |
| 4d. | In your opinions, do these services meet the standards in terms of;  Accessibility  Affordability  Availability  Quality |
| 4e. | In your opinions, are these services helping the intended people? |
| 4.f | In your opinions, what are the challenges faced by people who need to get these services? |
| 4.g | In your opinions, what recommendations would you give to improve on these services? |

**We have come to end of this discussion; we thank you for taking time to participate in this focus group discussion.**

**End**
