# Supplementary material for: Realising sexual and reproductive health and rights of adolescent girls and young women living in slums in Uganda: a qualitative study
Source: Reprod Health. 2021 Jun 12;18:125. doi: 10.1186/s12978-021-01174-z (PMC8199558; doi:10.1186/s12978-021-01174-z)
Supplement: Supplementary file 4 — Additional file 4. Is a Table outlining themes and sub-themes identified with illustrative quotes. [file 12978_2021_1174_MOESM4_ESM.docx]

**Additional File 4: Themes and sub-themes identified with illustrative quotes**

| Theme | Sub-theme | Quote |
| --- | --- | --- |
| Understanding of Sexual Health including sexually transmitted infections and HIV | Myths and misconceptions | “But they are fed the information like if you use a condom, it will get stuck inside you, you understand.” **Community Support Officer 1** |
|  | Knowledge of HIV among adolescent girls and young women (AGWY) | “I know that if you are infected, you can get medication and you stay alive” **Number 10,** **Focus Group 1** |
|  | Barriers to education provision | “They have the right but our government and leaders like the religious leaders currently don’t accept or allow these young girls to know about some health issues for example: reproductive health.” **Community Support Officer 2** |
| Understanding of Sexual and Reproductive Health and Rights (SRHR) | Understanding of consent  preventing redress of rights | ““I have a right to consent to treatment or not (to refuse medication). If the doctor has told me she/he is going to inject me, I have the right to consent to it or refuse it in preference to tablets” **Number not given, Focus Group 1** |
|  | Understanding of health rights | “I have the right to go and consult a professional in health” **Number 7, Focus Group 1** |
|  | Lack of knowledge about SRHR | “…however as you know many are not informed. Many are not informed. They are in the slums hiding there, they are stigmatised. She doesn’t feel even adequate to even seek for health” **Community Support Officer 1** |
|  | Lack of knowledge as a barrier to healthcare | “It is because some of them are not informed that there is such a service in the health facility. So they see the facility but they don’t know what it offers.” **District Leader 2** |
|  | Understanding of SRHR by stakeholders | “Right to health, right to friendly services and right to proper medication and treatment in that line. Those are the core ones in my heart.” **Community Support Officer 5** |
|  | Sources of knowledge for AGWY | “I think as a district we are running a radio program, every Thursday. So they can take advantage. They can take advantage of the radio program to disseminate more information about the services, printing out more IEC materials, which you can distribute through our systems and let them inform us formally about the operations on the ground. They can use the district as a medium to pass on the information” **District Leader 1** |
|  | Sources of knowledge for stakeholders | “I learnt of those rights from the different trainings we go to and different workshops we hold.” **Number 1, Focus Group 5** |
| Sources of support | Support whilst menstruating or sick | “I asked my older sister” **Number 2, Focus Group 1** |
|  | Disintegration of family system of support | “When boys and girls are growing up, they have entitlements, ideally some of this information should be passed on by their parents or extended family-aunties and the like. But the dis integration of the family system due to several factors has made this impossible.” **District Leader 1** |
|  | Role of peers | “May be to add, most of the violation i have been seeing is that some AGYW first run to their friends because they have them there. They have friends they first tell and them later advise them that let us do like this. So sometimes even these friends that we make have played a big role in helping in these issues.” **Number 6, Focus Group 3** |
|  | Source of support in case of harassment | “Most of them, when their rights are violated, they go to their friends, as you know anyone who has a friend they trust. Now, when you look at 15, 16, 17 years, if she has a relative whom they trust, sometimes they confine in them. The downside is, if they talk about it with their friends it may just stop there and nothing is done.” **Number 4, Focus Group 5** |
|  | Where to go for justice | “One of them is the local council 1 (Nabakyala). You can report your information can they can follow it up. They can either refer you, report it to police but they are there.” **Community Support Officer 4** |
| Experience of healthcare | Respect needed to improve healthcare | “What I want the doctors to change is that they should respect every patient despite the age differences even though a girl got pregnant at a tender age she should be respected so that she’s given the morale to be able to come back for antenatal.” **Number 5, Focus Group 2** |
|  | Positive experience of healthcare | “I was given a lot of care where I gave birth from even when I hadn’t paid money but that was in the [name omitted] government hospital.” **Number 10, Focus Group 2** |
|  | Needs unrecognised among AGWY | “I recommend that doctors first check before they diagnose what you are suffering from because sometimes you explain where the pain is, they tell you have candida yet sometimes you have ulcers and they give you medication that doesn’t work” **Number 4, Focus Group 1** |
|  | Different treatment if poor | “Yes they do respect the rights that’s if you’re financially stable unlike if when you don’t have the ability to pay for the services you can even give birth from the floor.” **Number 11, Focus Group 2** |
|  | Use of non-traditional medicine due to fear or cost | “They fear if they get a problem may be they want to abort, they can use these herbs and someone calls you and says this one is here dying, they say she has taken herbs.” **Community Support Officer 3** |
|  | Lack of resources or medications | “Then sometimes, also supplies are a challenge. We get drug stock outs here and there and it becomes a challenge for us to offer a service without stock.” **District Leader 2** |
|  | Lack of psychological support | “Very few, who come in a situation of seeking help or being counselled, what we would consider psychological support.” **Number 4, Focus Group 5** |
|  | Poor service at hospital | “You can fall sick and go to the government hospital thinking that you will be helped because we know medication there is free but you leave without receiving any medical treatment and they tell you there is no medication. Then they tell you to go to the pharmacy to buy medication and you don’t have the money so you leave without being helped” **Number 3, Focus Group 1** |
| Age, maturity and legal age | Age at maturity | “I got to know of my rights as soon as I turned 14yrs of age but understood about my rights.” **Number 11, Focus Group 2** |
|  | Being treated differently due to age | “in some government hospitals when you are pregnant and around the age of 15-16 they don’t first check if you can have a normal birth they just perform a caesarean section on you.” **Number 1, Focus Group 2** |
|  | Stigma due to age | “men stigmatizing us throwing comments such as look at her she gave birth at a young age look at her legs those are the comments they throw at us.” **Number 4, Focus Group 2** |
|  | Education needed to support young people | “Okay, I will start with schools, let information be made available to girls and boys as early as possible, perhaps in P3 or P4, let the information start and then grow gradually because girls and boys now know things at 5 or 6 years” **Community Support Officer 4** |
| Violations of rights and context | People with money are treated differently | “I was beaten badly on a certain tree as I tried to escape I was gang raped .When the authorities went to arrest them since they are financially stable they told the officers that I asked for it” **Number 4, Focus Group 2** |
|  | Attempted corruption | “my uncle came home and he was kind of forcing me to accept money from him which was 500,000shs and placed it on the table asking me to get the money and testify in court saying I wanted what happened to me” **Number 4, Focus Group 2** |
|  | Violations within healthcare | “In the hospital you’re looked at as a nasty thing that GOD doesn’t take care of saying I wonder who raped her come and I just work on you” **Number 4, Focus Group 2** |
|  | Breach of confidentiality | “what has brought the biggest issue is health worker, they don’t keep confidentiality.so people will not go to see them if they don’t keep confidentiality.” **Number 7, Focus Group 4** |
|  | Stigma associated with violation | “Then the shame that is attached to the person who had been violated, oh dear, society will be like “So she thought that reporting will help?” Everywhere she will pass, she will be despised the more.” **Community Support Officer 4** |
|  | Normalisation of sexual assault | “Even when it is committed or a crime of violence is committed, it is as abnormal and doesn’t overwhelm society for example; someone’s daughter has been raped, the first thing they would ask is “how old is the girl? 16 years.She is even at the age to marry.” They don’t consider this innocent girl, was she even willing? Is that what she wanted for her life? How is she going to deal with it for the rest of her life?” **Community Support Officer 4** |
|  | Taking advantage of AGYW | “Rape and defilement is one of them [challenges] and for some that have completed tertiary education, universities and the like, in the course of searching for jobs, people tend to take advantage” **District Leader 1** |
|  | Poverty as a driver for rights violations | “First of all, one of them is poverty, as you know these adolescents, they are people who want good life yet they don’t have the funds because sometimes their parents are not able to provide and because these elder men have the funds, they tend to be lured into those sexual practices.” **District Leader 3** |
|  | Power imbalance as a driver for rights violations | “Sir, when we go to child abuse, there are men who use their children/incest. A man tickles his childand she will never say it because he has to give her school fees and food. So in that way, they don’t get justice. I even wonder because there is a story I heard a story that a man produced a child and had sex with her and told her never to speak out. So we should not put the blame on only women, there are also men who are mistreating their daughter” **Number 9, Focus Group 3** |
| Barriers to justice | Barrier to justice Prioritising reputation | “But when I tried to tell the owner of the school, he told me to leave that alone and keep silent. [He said] “The good thing you are soon finishing senior 4 and you leave the school, leave that alone, because if you report, you are going to tarnish the school’s reputation.”” **Number 4, Focus Group 1** |
|  | Barrier to justice: Cost/corruption | “Violation of rights is present and common for example an older man can defile a young girl, the case proceeds to the police and all of a sudden, it disappears because of corruption” **Number 8, Focus Group 5** |
|  | Experiences of corruption | “Our side at the police, our mother had 100,000 ugx but she told them she had 50,000 ugx, then they agrees to come and arrest the person. But we waited for them and they didn’t show. When we went back, they told us we must add more money because what we had given them was less/little, so we abandoned the case. I advised my mother to leave the case because we didn’t get what we wanted, so we withdrew the case” **Number 6, Focus Group 1** |
|  | Barrier to justice: Stigma | “they don’t access the justice because one; when it so happens one is in that state that someone she is pregnant, found having sex, they are unfairly treated, chased away from home, denied their rights, they are discriminated, they are stigmatized right from their homes, families.” **Community Support Officer 6** |
|  | Health professionals not testifying following rape | “So what happened is that they refuse. They say now I signed for you, I am called to court, my transport and it happens. Sometimes they call them so often and they are not paid promptly every time you go so some tend to ignore so majority refuse to sign those forms to confirm yet the person sees the person may be has been raped” **District Leader 3** |
|  | Threats from violators | “Sometimes, it’s those exact people who are engaged with the AGYW who threaten them that if them that if they say anything it will be bad.” **Number 2, Focus Group 4** |
|  | Concern from victim they will be left without support | “So someone may not get justice because the man used her and that if the man is arrested, she may not get supports and there will be no one to support her. So it ends that it is like they have not got justice because she wants to find a way of ensuring the man looks after her.” **Number 9, Focus Group 3** |
|  | Police not doing their job properly | “Most of the time police does not do their work very well.” **Number 6, Focus Group 3** |
|  | Only school attendees taken seriously | “…government looks at defilement only looks at school going,a pupil or student who is affected that is when defilement is mentioned but there are these teenage mothers who are defiled by their parents, these maids who work in peoples homes are defiled by their bosses because but because they are not going toschool, the focus is not there, it’s not taken as an issue…” **Community Support Officer 6** |
| Role of parents | Parents forcing child marriage | “…our problem is with the parents who force their children to get married fast mainly because the parents too got married early.” **Number 5, Focus Group 4** |
|  | Parents do not believe in family planning | “There are parents that don’t believe in family planning. They come and say, my child has started taking medication. It’s also a problem especially because the parents also don’t know about the importance of family planning.” **Number 4,** **Focus Group 4** |
|  | Parents not open with children | “But most parents keep things secret from their children and we don’t tell them what is going on in the world.” **Number 5, Focus Group 3** |
|  | Belief that children will copy parents’ behaviours | “the way we sleep, people have started to engage husband and wife boy and girl, they sleep in one room and they engage in sex and children see and hear these things. This has brought out challenges that children have started engaging in these things because they what their parents do at night.” **Number 7,** **Focus Group 3** |
|  | Home environment as a driver for violation | “Most times, homes disintegrate, normally step mothers come into play and when they find children, they mistreat them which quickens or forces them to engage in sexual intercourse and they justify earning in situations of helplessness and they take that path.” **Number 6, Focus Group 5** |
|  | Money as a driver for child marriage | “Poverty, a parent may there with many children and there is one who has grown older and he gives her to a man to take because he cannot take care of her.” **Number 8, Focus Group 5** |
| Services available | Services available for HIV | “…here at the drop-in center we do carry out HIV self-testing and HIV testing services. And once one is found HIV positive we refer and do a linkage and follow up there at the health facility of her choice.” **Community Support Officer 3** |
|  | Services available from interviewees | “We do this through provision of sexual reproductive health or linkage, we link you for access to justice, or legal aid. We do economic empowerment, we do capacity building, we do research and documentation, education among others...” **Community Support Officer 7** |
|  | Traditional healers | “Sometimes they go to traditional healers, they consult them about a pregnancy, and they advise them to look for such and such herbs to terminate the pregnancy.” **Number 8, Focus Group 5** |
| Barriers to healthcare access | Lack of husband’s presence as a barrier to access | “Other hospitals when you go for antenatal without your husband they don’t understand they chase you away.” **Number 6, Focus Group 2** |
|  | Fear as a barrier to healthcare | “I took medicine from Kileku health centre but the nurses were so rude and tough I was afraid of them so I decided to run away.” **Number 10, Focus Group 2** |
|  | Distance as a barrier to healthcare | “…the distance is long and it is not like everyone can walk there or has the money…” **Number 10, Focus Group 1** |
|  | Stigma as a barrier to access | “So because of that they shy away from accessing the service because they fear to be stigmatized and abused” **Community Support Officer 6** |
|  | Husband as barrier to access | “…their husbands don’t want them to take family planning, and you may find that once she gets pregnant she aborts in secret. She doesn’t want the husband to know, you find there is even cruelty in the homes where they are married.” **Community Support Officer 3** |
|  | Parents as a barrier to access | “There are parents that don’t believe in family planning. They come and say, my child has started taking medication. It’s also a problem especially because the parents also don’t know about the importance of family planning.” **Number 4, Focus Group 4** |
|  | Cost as a barrier to access | “I went to a government hospital and I didn’t receive any medical treatment, when I went to the clinic, I was told that I first have to pay to receive any kind of medical attention and the money was a lot of which I didn’t have” **Number 3, Focus Group 1** |
|  | Language as a barrier | “First of all the AGYW don’t know what family planning and in many cases they are in English and many of them don’t know English and they don’t know when to use them and at what stage to take them.” **Number 7,** **Focus Group 4** |
|  | Rumours as a barrier to care | “So those rumours from the community brings problems, one incident tarnishes the reputation of the health centre which would not have been the case. I would think if people stopped rumours because it can prevent people from getting medical help in time.” **Number 2, Focus Group 5** |
| Consequences of pregnancy | Thrown out due to pregnancy | “Then have had cases of denial of rights like staying at home, cases of adolescents who have been thrown out of home because they have got pregnant,” **Community Support Officer 6** |
|  | School dropout due to pregnancy | “One of them is that AGYW drop out of school-they get pregnant and drop out. When they get pregnant, they feel their future has ended, instead of thinking of going back to school after giving birth, they feel their life is wasted, they are no longer fitting in the community,” **Number 12, Focus Group 3** |
| Drivers for violations | Alcohol and drugs as driver for violations | “Sexual domestic violence is high in areas like this. For us men, the rate of drinking alcohol and drug abuse-you know what theses result into. When they go back home, they harass women, because of poverty and other things.So our sisters have got problems because domestic violence is high and rape” **no number given, Focus Group 3** |
|  | Belief that AGWY dressed inappropriately | “In the past, people used to dress well. But these days, you find a girl or even a boy dressed with all the private parts outside. This attracts men to rape girls but also boys are also raped based on the way they are dressed. Even boys dressing code attracts women.” **Number 1, Focus Group 3** |
|  | Money as a driver for exploitation | “Someone just lies the child with little money and then rapes her. So someone will tell them ‘’you give me and I give you money’’. They will entice and attract them with money and then rape them. The girls don’t even ask about the state of where they are going. Even the youngest child will ask for money. I don’t know whether the world has change” **number 12, Focus Group 3** |
| Redress of rights and challenges | Informal redress of rights | “The other challenge that we have is that parents also always negotiate with the people that rape their children and we find ourselves in situations where cases are settled between families without consideration of the challenges that the survivor is going through or even worse still the diseases she many contract from such experiences.” **Number 5 Focus Group 4** |
|  | Delay in redress | “Then the other one is the length of attaining justice, even when the police is willing to take up the case, the justice system delays. It is not easy.” **Community Support Officer 4** |
|  | Length of sentence felt to be too short | “Can I also add that the police is not doing us enough justice because ideally whoever violates should be handled by a strong law, people don’t understand that once you violate a girl’s or boy’s sexual rights you are ruining this person for good. Someone found guilty takes a very light sentence and goes and violates another one, they bring them back and it’s the same light sentence.” **Community Support Officer 4** |
| Sources of information regarding SRHR and services and areas for improvement / challenge | How AGWY know about service availability | “I have never seen these services but I just see a car pass by looking for those who need the services” **Number 6, Focus Group 1** |
|  | Need to educate boys as well as girls | “So I think the other challenge is sexual and reproductive health rights being looked at as exclusively for girls, so the boys will always do whatever they want thinking that this is only for girls, which to me is something the society needs to come out strongly to educate the male youths.” **Community Support Officer 4** |
|  | Misconceptions among stakeholders | “Maybe the issues with family planning country wide is that when using family planning it someone’s blood samples should be taken to establish the blood group and what kind of medication the person needs. To add to what the lady has just said, may people only go to clinics and get what is it called, slips! Pills! Without getting to know which medication their blood group should have. As a result we are seeing many emerging issues of cancer, wounds in the stomach, leading to poor health conditions among the AGYW. Simply because they are not well educated on what type of medicine they should be taking as people of different age groups.” **Number 4, Focus Group 4** |
|  | Resistance to learning among AGYW | “Most don’t want to be sensitized. In that even though you organize a training, they do not attend and yet they would have been of use to them in learning and understanding if there is any chance of an issue like this happening in the future and it is in these trainings where they would get knowledge on what to do in case the need arises.” **Number 6,** **Focus Group 5** |
|  | Slum context for learning | “Being that in the slum we have a lot of diseases and that is why you see that most problems breakout amongst us, because we are in a congested environment and it is easy to mingle with one another to worsen the situation. And what can help us is to increase and emphasize trainings and sensitization, even though we sensitize five and another ten in order to reduce the current situation and improve attendance in sensitization.” **Number 6,** **Focus Group 5** |
|  | Feelings that AGYW will not do as told | “…those AGYW are very hard to deal with simply because everything you stop them from doing they think you’re against them. For instance when she gets pregnant before 18, she can’t admit that the timing is wrong…” **Number 5, Focus Group 4** |
|  | Need for education | “The other recommendation is more community awareness is needed, very intense, actually intense, so that the adolescent girls can be equipped with information regarding SRH and HIV and they are told on what to ask for, what to expect.” **Community Support Officer 7** |
|  | Need to advertise services more | “…they don’t keep informing the community of the availability of these services and the fact that Wakiso is a peri-urban district, people settle and people go away people settle and people go away. So the people who come in tend to miss the services thinking they are not available.” **District Leader 1** |
| Changes needed to allow redress of rights | Need for empowerment | “If they are empowered enough. May be they can begin the talk perhaps they don’t have enough capacity to get up and talk about these violations you know that they even need that. If they have the empowerment. Enough empowerment, the sources they can stand up to this within that talk.” **Community Support Officer 7** |
|  | Government needs to change policy/law | “They may want to acquire post abortion or abortion services but its against the law so they don’t, may be some of these cases end up into death” **Community Support Officer 5** |
| Suggestions for services | Training for stakeholders | “I would recommend, that they should increase training of health workers, counsellors because more than not counsellors are the first point of contact for these people who have come to the hospital and they are the ones who must inform the person about the HIV status and help the person accept the reality of the situation.” **Community Support Officer 2** |
|  | More funding | “More funding should be directed towards Adolescent health because we really don’t have a vote for adolescent health as a standalone. We are running ….integrating it with other services and we run on other service now like HIV and ride on them.” **District Leader 2** |
|  | Stop corruption | “government needs to fight corruption. Corruption is a very big problem in our community so the government will need to come out very strongly in this area.” **Number 7, Focus Group 4** |
|  | Increase service availability | “for me I encourage government to increase health facilities in communities as this will make them even more accessible to the people” **Number 6, Focus Group 4** |
|  | Empower women | “I think the government of Uganda should empower women as the biggest stake holder in this issue. They should really empower the women.” **District Leader 2** |
